# Hepatoprotective Potential of Some Local Medicinal Plants against 2-Acetylaminoflourene-Induced Damage in Rat

**DOI:** 10.1155/2013/272097

**Published:** 2013-09-15

**Authors:** Adewale Adetutu, Olubukola S. Olorunnisola

**Affiliations:** Department of Biochemistry, Faculty of Health Sciences, Ladoke Akintola University of Technology, P.M.B. 4000, Ogbomoso, Nigeria

## Abstract

The *in vivo *micronucleus assay was used to examine the anticlastogenic effects of crude extracts of *Bridelia ferruginea, Vernonia amygdalina, Tridax procumbens, Ocimum gratissimum, *and *Lawsonia inermis *in Wistar albino rats. Extracts of doses of 100 mg/kg body weight were given to rats in five groups for seven consecutive days followed by a single dose of 2-AAF (0.5 mmol/kg body weight). The rats were sacrificed after 24 hours and their bone marrow smears were prepared on glass slides stained with Giemsa. The micronucleated polychromatic erythrocyte cells (mPCEs) were thereafter recorded. The hepatoprotective effects of the plant extracts against 2-AAF-induced liver toxicity in rats were evaluated by monitoring the levels of alkaline phosphatase (ALP), gamma glutamyl transferase (GGT), and histopathological analysis. The results of the 2-AAF-induced liver toxicity experiments showed that rats treated with the plant extracts (100 mg/kg) showed a significant decrease in mPCEs as compared with the positive control. The rats treated with the plant extracts did not show any significant change in the concentration of ALP and GGT in comparison with the negative control group whereas the 2-AAF group showed a significant increase (*P* < 0.05) in these parameters. Some of the leaf extracts also showed protective effects against histopathological alterations. This study suggests that the leaf extracts have hepatoprotective potential, thereby justifying their ethnopharmacological uses.

## 1. Introduction 

There is a huge patronage of herbal products around the world as an alternative to orthodox drugs [1, 2] and these medicinal plants have immensely contributed to the development of human health and welfare [3, 4]. Previous studies on several folklore herbs showed that plant extracts contain many compounds with chemoprotective potentials that may prevent the attack of carcinogens [5, 6]. However, limited studies have investigated the anticlastogenic potentials of African herbs used in folk medicine to treat cancer.

Various test systems have been used to evaluate the protective effects of plant extracts against genotoxicity induced by carcinogens [7–9]. Genotoxic carcinogens, including 2-AAF, often cause a variety of nongenotoxic alterations in cells which might be indispensable in tumorigenesis. Various studies used different models to investigate the effect of herbs on alleviating oxidative stress on the liver [3]. Carcinogenic 2-AAF was selected in this study because of the ability to induce chronic liver toxicity and tumors in a number of species in the liver, bladder, and kidney [7, 10]. Therefore, this study was aimed at investigating the hepatoprotective effects of *B. ferruguinea, V. amygdalina, T. procumbens, O. gratissimum, *and* L. inermis* extracts against 2-AAF-induced toxicity in rats.

## 2. Materials and Methods

### 2.1. Plant Materials and Extraction

The leaves of the selected plants were collected in February 2011, from the Ladoke Akintola University of Technology (LAUTECH) Farm, Ogbomoso, Oyo, State Nigeria. The authentication of the plants was carried out by Mr. T. K. Odewo in the Herbarium of the Forest Research Institute (FRIN), Ibadan, Nigeria, where the voucher specimens were deposited. The voucher numbers assigned are *Ocimum gratissimum, *FHI. 107392; * Vernonia amygdalina, *FHI. 107399; *Tridax procumbens, *FHI. 107397; *Parkia biglobosa,* FHI. 107395; and *Bridelia ferruginea, *FHI. 107393. The leaves of the plant materials were air dried for 3 weeks until a constant weight was obtained and ground into powdered form. 100 g of the ground leaves was macerated in 500 mls of distilled water at 4°C under refrigeration, for three days with occasional agitation. The aqueous extracts were then filtered through Whatman No.1 filter paper and the filtrate was concentrated using a freeze dryer. Healthy Wistar rats with an average weight of 188 g were obtained and housed in the preclinical animal house, Faculty of Basic Medical Sciences, LAUTECH, Ogbomoso. The rats were kept five per cage with hust bedding and fed with pellets (obtained from Irorun-Agbe feed mills, Ogbomoso) and water *ad libitum*. This study was conducted in accordance with the Applied Ethics in Animal Research and it was approved by the Ethical Committee of College of Medicine, LAUTEC H, Ogbomoso, Nigeria.

### 2.2. Experimental Design and Micronucleus Assay

Rats were randomly divided into 7 groups of five animals each. The plant extracts were orally administered at the dose of 100 mg/kg body weight for seven consecutive days followed by a single dose of 2-AAF (0.5 Mmol/kg body weight) which was administered intraperitoneally. The rats in the negative control group were given distilled water while those in the positive control group received 2-AAF. The rats were sacrificed after 24 hours and their bone marrow was flushed and smears were prepared on glass slides. These were stained with Giemsa and viewed under a microscope and a tally counter was used to record the frequency of micronucleated polychromatic erythrocyte cells (mPCEs). 

### 2.3. Serum Preparation, Enzyme Assay and Liver Isolation

The rats of each group were anaesthetized with ether, and blood was collected directly from the heart. The blood was centrifuged at 2,000 g for 10 min at 4°C to separate the serum and kept at 4°C to assay the activities of the serum enzymes. Alkaline phosphatase (ALP) and gamma glutamyltransferase (GGT) were determined using assay kits (Labkit, Spain) according to the manufacturer's instructions. The liver was removed carefully after sacrificing the animals by cervical dislocation. The livers were fixed in 10% buffered formalin and then dehydrated in a graded series of alcohol, cleared in xylene, and embedded in paraffin wax. Multiple 5 *μ*m sections from each block were mounted on slides and stained with hematoxylin-eosin dye. 

### 2.4. Statistical Analysis

All values are mean ± S.E.M. (*n* = 5). For statistical analysis, one-way ANOVA with Duncan's variance (SPSS 15) was used to compare the groups. In all the cases, a difference was considered significant when *P* < 0.05.

## 3. Results

### 3.1. Micronucleus Test and Enzyme Assay

The rats administered with 2-AAF alone showed relatively high values of mPCE frequency, ALP and GGT concentrations when compared to the negative control group (Tables 1 and 2). For the groups administered with crude plant extracts followed by 2-AAF (groups A–E), there was a significant decrease in the mPCE frequency (Table 1) and enzyme concentrations (Table 2) as compared to the negative control group. Similarly, in the oxidative injury experiments, the negative control group demonstrated relatively low values of ALP and GGT concentration, while the 2-AAF-treated group showed elevated levels of ALP and GGT (Table 2). The biochemical parameters of the extract-treated group were higher than those of the control group (*P* < 0.05), but it showed much lower levels of ALP and GGT than the 2-AAF treated group.

### 3.2. Histopathology Analysis of the Rat Liver

**Figure 1:**
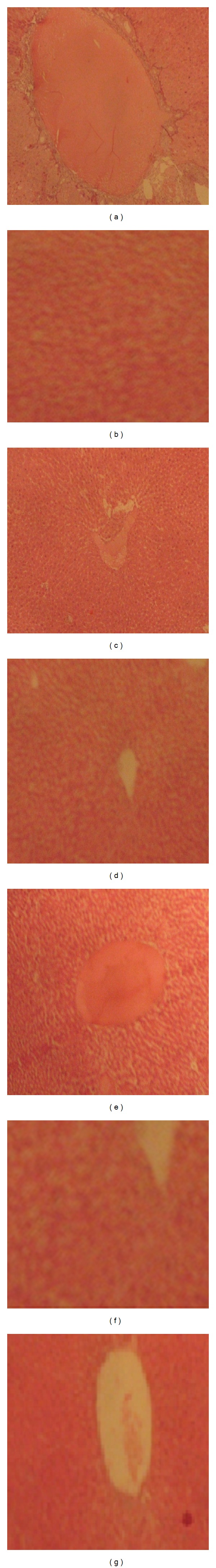
Representative photomicrographs of H&E stained sections of the liver of the rat fed with 2-AAF and plant extracts at 200X magnification. (a) Photomicrograph of the liver of the rat fed with 2-AAF only. (b) Photomicrograph of the liver of the rat fed with distilled water only. (c) Photomicrograph of the liver of rat fed with *B. ferruguinea *and 2-AAF. (d) Photomicrograph of the liver of rat fed with *V. amygdalina *and 2-AAF. (e) Photomicrograph of the liver of rat fed with *O. gratissimum* and 2-AAF. (f) Photomicrograph of the liver of rat fed with* L. inermis* and 2-AAF. (g) Photomicrograph of the liver of the rat fed with *T. procumbens* and 2-AAF.

Histopathology experiment was carried out on the liver of the rats fed with plant extracts and 2-AAF and the results are shown in Figures 1(a)–1(g).  Figure 1(a) shows the liver of 2-AAF only treated rats. The liver sections of these rats showed necrosis across the cells and erosion in hepatic plates and loss of cellular margins. The hepatocytes are disordered and sinusoids are damaged as well. In the negative control group (Figure 1(b)), the cells are normal in shape, the cell nuclei are intact, and, most notably, the portal vein has a regular shape and healthy set of cells can be observed. In the rat treated with plant extracts and 2-AAF, the histological architecture of treated liver sections revealed a mild degree of degeneration and necrosis with the hepatocytes nuclei at a recovery stage (Figures 1(c)–1(g)). 

## 4. Discussion

The growth of cancer has been seen as a progressive multistep development of transformation of normal cells into malignant cells motivated by genetic alterations that include mutations in tumour suppressor genes and oncogenes and chromosomal damage [11, 12]. This study evaluated the protective effects of five popular medicinal plants against 2-AAF-induced chromosomal damage and hepatotoxicity. These plants were selected basically because of their popular use to treat various diseases in Africa including cancer [13–15]. 

In this study, the selected plant extracts produced no clastogenic effect with respect to micronuclei frequency in rats' bone marrow. However, the 2-AAF-treated group confirmed an indication of chromosomal damage with increase in number of mPCE as compared with the negative control group. Similarly, the serum ALP and GGT level evaluation confirmed that 2-AAF caused liver injury at the doses injected into the rats. In addition, the elevation of cytoplasmic ALP and GGT is considered an indicator for the release of enzymes from disrupted cells.

Therefore, these results are in conformity with the previous reports that demonstrated that carcinogenic agents like 2-AAF produced sufficient injury to the hepatic parenchyma [16–18]. On the other hand, treatments with the extracts significantly reduced the levels of ALP and GGT compared to the 2-AAF-treated rats. The decrease in the serum levels of these enzymes might be due to the presence of various phenolic compounds in the leaf extract that enhanced the liver's regeneration ability [18]. 

Furthermore, the histopathological studies conducted on the liver of the treated rats revealed that the extract were effective in reducing the 2-AAF-induced histopathological lesions and almost normal liver architecture was exhibited, with well-formed hepatocytes separated by sinusoids and maintained cord arrangement. Consequently, the histopathological examination thus verified the hepatoprotective effects of the extract against the 2-AAF-induced hepatotoxicity. The protective effects exhibited by the selected plants in this study might be due to various interactions between the complex crude extracts and the mechanisms involved in 2-AAF induced toxicity. Reported antioxidant and anticancer activities and the phenolic content of the extracts may be responsible for their protective activity [13, 15, 19].

*T. procumbens* is used for various problems related to the liver such as jaundice, hepatitis, cirrhosis, and heart burn. It has been demonstrated that the chloroform insoluble fraction of ethanol extract of *T. procumbens *is most potent in alleviating the oxidative stress/liver injury caused by factors similar to viral hepatitis, drug intoxication, and lipid peroxidation due to reactive oxidative species [15, 20]. *O. gratissimum *has been used extensively in the traditional system of medicine in many countries. In the Northeast of Brazil, it is used for medicinal, condiment, and culinary purposes. In the coastal areas of Nigeria, the plant is used in the treatment of epilepsy, high fever, and diarrhoea. In the Savannah areas, decoctions of the leaves are used to treat mental illness [21, 22]. 

Topical application of *L. inermis* leaf extract at a dose level of 1000 mg/kg body weight was found to be effective in reducing the number of papillomas. Izevbigie [14] demonstrated that *V. amygdalina* might be a strong candidate for cancer management and that edotides may be the principles in *V. amygdalina *that are responsible for its anticancer activity. In addition, Jisaka et al. [19] had shown that the extract of *V. amygdalina *has potent anticancer activities and the flavonoid content of the extracts may be responsible for their activity. *B*. *ferruginea* leaves have been used to treat diabetes and as a purgative and a vermifuge [13]. The bark extract is being used for milk coagulation and also in lime juice for the formulation of traditional gargle “ogun efu” [23]. 

In the present study, *B. ferruguinea, V. amygdalina, T. procumbens, O. gratissimum,* and* L. inermis *leaf extracts demonstrated hepatoprotective potentials in a rat model of 2-AAF-induced hepatotoxicity. The hepatoprotective activity of the leaf extracts may be due to the free radical scavenging and antioxidant activity, resulting from the presence of some phenolic compounds in the extracts. Further studies are in progress to better understand the mechanism of action of the plant extracts responsible for the observed hepatoprotective activity.

## Figures and Tables

**Table 1:** mPCEs count in rats treated with the plant extracts and 2-AAF.

Groups	Treatments	mPCEs
A	*B. ferruguinea* + 2-AAF	6.0 ± 2.0
B	*V. amygdalina* + 2-AAF	10.5 ± 3.5*
C	*O. grastissimum* + 2-AAF	19.5 ± 8.5*
D	*L. inermis *+ 2-AAF	5.5 ± 1.5*
E	*T. procumbens *+ 2-AAF	13.0 ± 7.1*
F (ve control)	Distilled water	4.1 ± 0.02
G (ve control)	2-AAF	26.0 ± 7.2**

Results are expressed as mean ± S.E.M (*n* = 5); *statistically significant compared to 2-AAF treated animals (*P* < 0.05); **statistically significant compared to negative control animals (*P* < 0.05).

**Table 2:** ALP and GGT concentration in rats treated with the plant extracts and 2-AAF.

Group	Treatments	ALP (IU/L)	GGT (IU/L)
A	*B. ferruguinea* + 2-AAF	9.58 ± 2.94*	3.09 ± 0.01*
B	*V. amygdalina* + 2-AAF	5.21 ± 0.46*	6.14 ± 0.44*
C	*O. gratissimum* + 2-AAF	8.30 ± 3.21	5.24 ± 0.44*
D	*L. inermis *+ 2-AAF	8.5 ± 1.38*	6.01 ± 0.69*
E	*T. procumbens *+ 2-AAF	8.29 ± 0.01	3.09 ± 0.01*
F	Negative control	4.13 ± 0.01	2.98 ± 0.01
G	2-AAF	17.88 ± 2.29**	13.8 ± 0.36**

Results are expressed as mean ± S.E.M (*n* = 5); *statistically significant compared to 2-AAF treated animals (*P* < 0.05); **statistically significant compared to negative control animals (*P* < 0.05).
